# High-temperature tensile strength of C/SiC composite under laser-induced high heating flux in an aerobic environment

**DOI:** 10.1038/s41598-024-57266-w

**Published:** 2024-03-21

**Authors:** Jiawei Wang, Bin Li, Shanshan Wang, Shunlei Zhang, Pengling Yang, Chenghua Wei, Yanlong Shen

**Affiliations:** 1 College of Aeronautics, Northwestern Polytechnic University, Xi’an, 710072 China; 2 State Key Laboratory of Laser Interaction with Matter, Northwest Institute of Nuclear Technology, Xi’an, 710024 China

**Keywords:** C/SiC, Tensile strength, High temperature, Laser, Double-sided irradiation, Mechanical engineering, Composites

## Abstract

Based on the advantages of laser high brightness, a high-temperature mechanical property measuring device has been developed, which can measure the high-temperature strength of C/SiC composites under the condition of short-term high-temperature rise rate and solve the problem of over-oxidation of materials in conventional high-temperature mechanical properties experiments. The experimental results show that the maximum temperature rise rate is 260 ℃/s at the initial heating stage, and the test time is controlled within 35 s. The tensile strength of the prepared C/SiC composites decreased first and then increased at high temperatures and laser-induced high temperatures. The experimental results are similar to those in the literature under the inert atmosphere. Oxidation has less of an effect on the mechanical characteristics of materials under conditions of rapid temperature rise. The system can be used to test the mechanical properties of composite materials at high temperatures and as a simulation platform for the thermal response of specific thermal protection systems subjected to a constant heat flux. This study can provide a new idea for testing ultra-high temperature mechanical properties of C/SiC materials and provide key technical support for the engineering application and high-temperature testing of C/SiC materials in high-temperature environments.

## Introduction

The service safety of C/SiC composites in high-temperature environments is a major issue that must be resolved^1–5^. The development of a new generation of high-temperature mechanical testing technology for ultra-high-temperature composites is of critical importance. Conducting high-temperature mechanical property experiments in an aerobic environment is now the greatest obstacle to the advancement of thermal protective material technologies^6,7^. The main heating methods for high-temperature mechanical properties of materials are environmental heating, induction heating and electric heating. The advantage of the environmental heating testing machine is that the heating is universal and the heating temperature is uniform^8–12^. However, the disadvantage is that the heating rate is generally slow (5–50 ℃/min), the material is easily affected by over-oxidation and creep in the process of temperature rise, and the performance is different from that in the real service environment, and the testing efficiency is low. In an aerobic environment, the maximum operating temperature is generally below 1700 ℃ due to the effect of oxidation on the heating body. This method of heating can easily result in the specimen’s over-oxidation over a lengthy heating phase, resulting in a significant divergence between the test results and the material's performance in a real-world service environment. Induction heating is an excellent method for evaluating the high-temperature mechanical characteristics of materials in an atmosphere. The advantage of induction heating is that the heating rate is high, and the heating rate of graphite as the heating body in an inert environment can reach 600 ℃/min^13,14^. However, the induction heating testing equipment requires the specimen to conduct electricity, and the heating has the issue of uneven temperature distribution, which will result in the occurrence of local overheating and influence the performance evaluation of the material. Due to the susceptibility of electrically heated electrodes to oxidation at elevated temperatures, this approach is mostly applicable in inert conditions. In a heating environment, it is necessary to prepare a sophisticated, water-cooled fixture with a particular design. In addition, the three aforementioned types of ultra-high temperature mechanical testing equipment must be outfitted with a particular water-cooling and fixture design and a complex structure. The ways in which to improve the heating mode of ultra-high temperature mechanical properties experiments is, therefore, a pressing issue that must be solved^15^. Laser has the advantage of high luminosity and the unique advantage of heating materials at high temperatures and at a fast rate. By heating materials with a laser, numerous types of in-situ measurements of materials have been made possible^16,17^. To date, however, there have been no published reports on the use of laser heating for assessing the mechanical properties of materials at high temperatures. According to the analysis, there are two reasons for this result: first, the traditional laser Gaussian spot centre has high light intensity and low edge light intensity, which causes uneven heating of the material under laser heating and makes it difficult to characterize the high-temperature mechanical properties of the material; and second, the output power of the laser is low, which cannot meet the heating demand in the high-temperature region. With the advent of fibre laser technology, these two issues have been effectively addressed. Currently, multi-mode continuous fibre lasers can produce a flat-topped beam of high kilowatts for an extended period of time, and a long-term uniform temperature field can be created on the surface of the material to heat it. It is possible to establish a method for assessing high-temperature mechanical properties using a flat-top fibre laser beam.

When the rate of temperature rise is slow, the specimen is susceptible to severe oxidation during the testing procedure, resulting in considerable discrepancies between the test findings and the real service environment performance of the material. This study conducted the experimental investigation of the tensile strength of C/SiC materials in a high-temperature environment based on laser-induced high temperature to address this issue. The thermal–mechanical coupling test method of high-temperature rise rate for various materials is studied, which provides technical support for further establishing the high-temperature rise rate ultra-high temperature mechanical property test system based on laser heating.

## Ultra-high temperature strength test of materials

### Preparation of experimental samples

**Figure 1:**
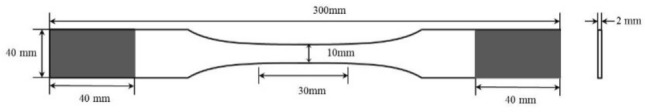
Needle-punched SiC composites used in the experiment.

The C/SiC composite materials used in this article are manufactured using the Chemical Vapor Infiltration (CVI) method. The fiber preforms are produced using T700 carbon fibers produced by Toray Corporation in Japan. They are made by stacking layers of short fiber nets and long fiber non-woven fabrics and needle punching them together. After the carbon fiber preforms are treated at high temperatures, they are placed in a SiC gas phase deposition furnace for infiltration. The infiltrated preforms are then cut to the desired size after being formed as a whole. The length of the specimen is 300 mm, and the thickness is 2 mm. The test section length is 30 mm. As indicated in Fig. 1, a 40 mm stiffener is attached to both ends of the specimen to prevent it from slipping under mechanical loading.

### Experimental system of high-temperature mechanics based on laser heating

**Figure 2:**
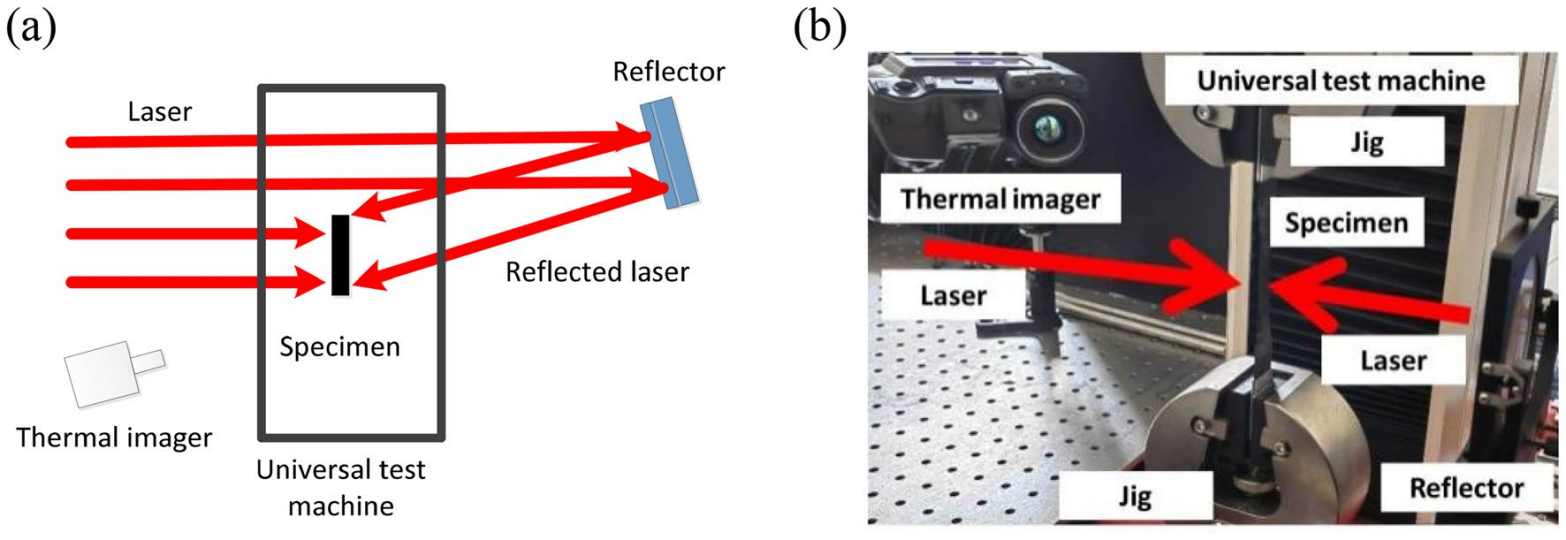
Experimental Layout Chart: (**a**) schematic drawings; (**b**) physical drawings.

Figure 2 shows an experimental system based on laser heating. Due to the advantages of laser heating's good orientation, heating does not require a heating chamber, and the entire testing environment is open and unobstructed. The heating laser uses a 1080 nm-wavelength fibre laser. The laser is able to maintain a consistent power output. The laser spot must be homogenised to attain a homogeneous specimen heating temperature. Through optical fibre bunching, the square flat-topped spot is generated in this paper. The C/SiC specimen is clamped on the fixture of the universal testing machine, and the uniform square laser spot of about 30 mm × 30 mm is irradiated on the surface of the specimen, which can cover the testing section of the specimen completely. Through full mirror reflection, a residual spot is burned on the back surface of the specimen, and the effect of laser irradiating the same region of the specimen from both sides is achieved. To assure safety, a graphite plate is positioned in the laser's path to absorb any surplus laser energy. An exhaust fan was positioned near the specimen to prevent the heating air of C/SiC during laser heating from interfering with the thermal imager's observation. The loading rate of the universal testing machine is 20 mm/min. A thermal imager recorded the temperature of the specimen under laser heating, and the emissivity of the thermal imager was set to 0.96 until the fracture test of the specimen was stopped under laser heating.

The tensile strength of the specimen can be expressed as

1 $$\sigma (t,T) = \frac{P(t)}{{bh}}$$

In the above formula, *t* represents the time when the specimen breaks, *T* is the temperature when the fracture occurs, *P* is the load when the fracture occurs, *b* is the width of the material, and *h* is the thickness of the material.

### Experiment and methodology

**Figure 3:**
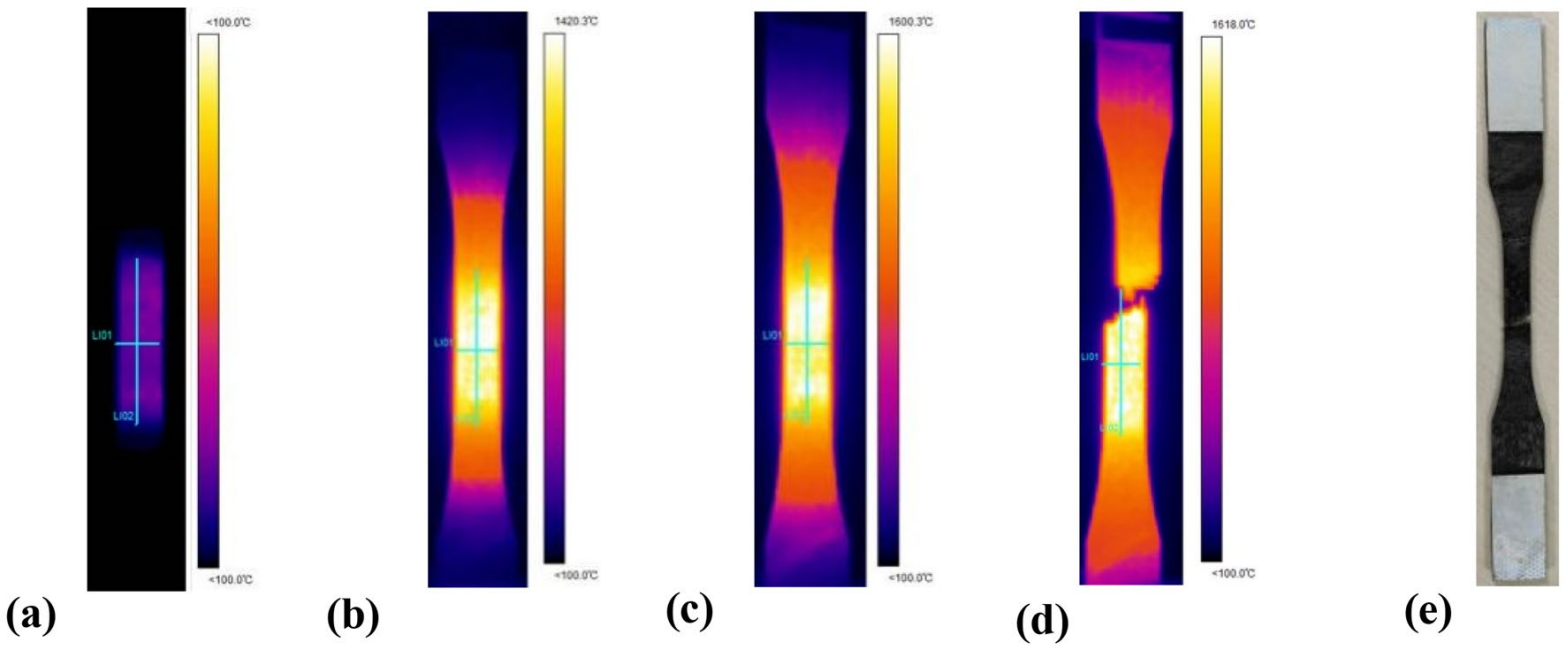
Experimental results recorded by thermal imager (laser heating power density 281.2 W/cm^2^). (**a**) Begin to shine, (**b**) Irradiation of 5s, (**c**) Irradiation of 10 s, (**d**) Moment of, (**e**) fracture.

The samples were irradiated with laser heating power densities of 37.1 W/cm^2^, 70.3 W/cm^2^, 140.6 W/cm^2^ and 281.2 W/cm^2^, respectively. At each laser power density point, the experiment was conducted three times. The typical experimental findings captured by the thermal imager are depicted in Fig. 3. To examine the temperature uniformity within the laser heating region, two center lines (L01 and L02) are drawn on the thermal image of the laser heating region. L01 represents the horizontal temperature at the center of the laser heating region, while L02 represents the longitudinal temperature distribution at the center of the laser heating region. The results indicate a slight thermal expansion in the transverse and longitudinal directions of the specimen under laser heating; the central temperature of the specimen increases continuously to the test temperature, and the infrared image reveals a more uniform temperature distribution in the test section. The final fracture of the specimen is located near the centre of the test area. When the temperature of the test section reaches about 1600℃, the position temperature between the two ends of the material and the fixture is always below 100℃. Therefore, this experimental apparatus can detect high-temperature mechanical properties without the addition of any water-cooling apparatus. The structural design is straightforward compared to previous high-temperature mechanical test systems developed using the heating method.

## Results and discussion

**Figure 4:**
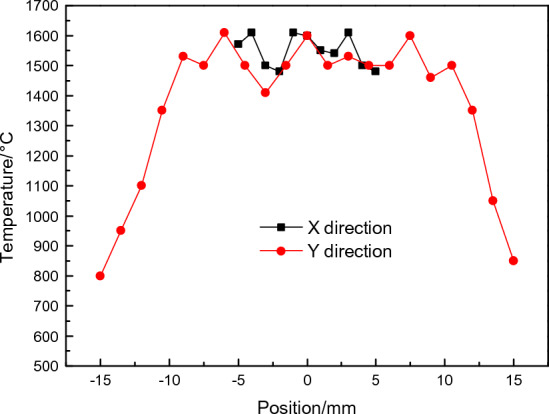
Temperature distribution of specimen in transverse and longitudinal direction.

Figure 4 shows the temperature line distribution of L01 and L02 in transverse and longitudinal straight lines with a laser heating power density of 281.2 W/cm^2^. The results show that the transverse temperature distribution of the material is relatively uniform, the maximum temperature distribution error is about 100 ℃, the longitudinal temperature distribution is affected by heat conduction, and the temperature distribution is different. However, the temperature distribution is more consistent in the 10 mm range of the most central region, with a maximum temperature difference of 100 °C. As a result, the average temperature within 10 mm10 mm of the heating centre region of the specimen is regarded as the test temperature's defining characteristic. In the experiment, the mean temperature in the 10 mm × 10 mm at the centre of the test area was taken as the characteristic of the test temperature.

**Figure 5:**
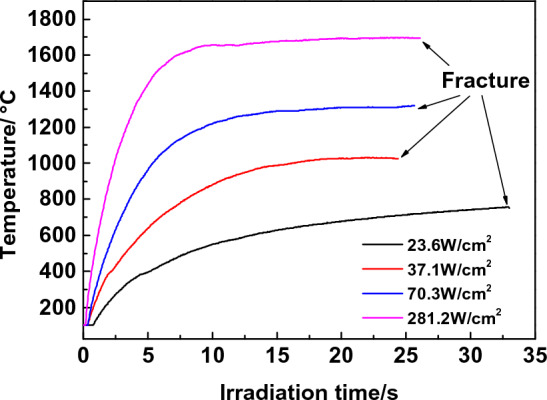
Laser double-side irradiation heating C/SiC material scene.

The centre maximum temperature recorded by the thermal imager under varied laser heating power densities during laser heating is depicted in Fig. 5, together with the temperature distribution along the specimen's centre transverse and longitudinal lines. In the early phase of laser heating, the material will be rapidly heated, but the temperature will shortly reach thermal equilibrium, and temperature fluctuations will be minimal when the specimen finally breaks. Fig. S8 shows the typical temperature distribution along the centre transverse and longitudinal direction of the specimen after the temperature of the specimen reaches a steady state under laser heating recorded by the thermal imager. The mean temperature in the 10 mm × 10 mm zone at the centre of the fracture position was measured when the specimen was fractured. In the experiment, the temperature rise rate of the specimen before heating was about 70 ℃/s, 110 ℃/s, 180 ℃/s, 260 ℃/s, and the temperature rise in the beginning 5 s was about 37.1 W/cm^2^, 70.3 W/cm^2^, 140.6 W/cm^2^, 281.2 W/cm^2^, respectively. Until the specimen’s final entry into equilibrium temperature, the specimen's surface temperature is constant, and the temperature change is minimal when the fracture occurs.

**Figure 6:**
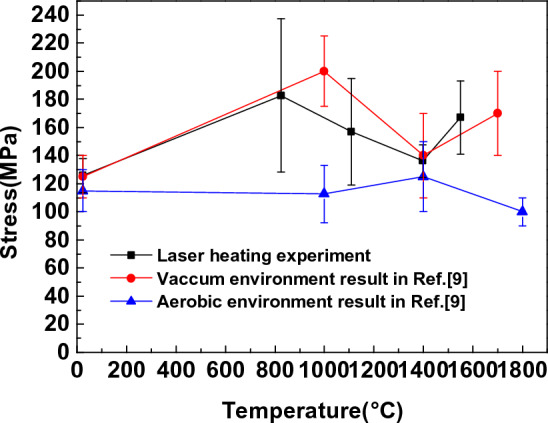
High-Temperature Tensile Strength of C/SiC Composites.

**Figure 7:**
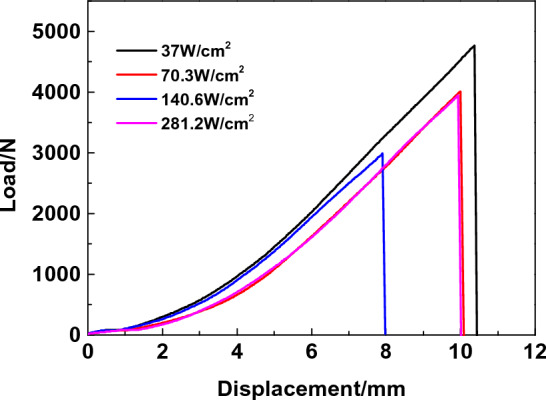
Typical load–displacement curves of C/SiC obtained by experiment.

Figure 6 shows the variation law of high-temperature tensile mechanical properties of C/SiC composites with temperature measured by experiments. The results indicate that the tensile strength of C/SiC composites in an aerobic environment increases with temperature first, then falls, and then increases again. The high-temperature tensile strength of C/SiC materials under an anaerobic environment is given by comparison with the Ref.^9^. The two materials have the same trend. The analysis reveals that oxidation has less of an effect on the material due to the rapid rise in temperature and the short duration at high temperatures, which is less than 35 s. The results are comparable to those in an anaerobic environment. Under the laser-induced high-temperature rise, the material reaches the desired temperature within a few seconds, the time of oxidation is brief, and the factors affecting the sample are minimal, so the experimental results are comparable to those in the inert atmosphere. In addition, the tensile strength of C/SiC composites is affected not only by temperature dependence and residual thermal stress but also by the fracture mode and mechanism. For T700 carbon fibre, the tensile strength increases slowly at the initial stage under the influence of high temperature and then decreases with the increase in temperature. Prior to 1500 °C, the tensile strength of bulk SiC materials increases as the temperature rises. Therefore, the material's maximal strength looks to be approximately 1000 °C. When the temperature rises higher, carbon fibre oxidation occurs, and its strength decreases, leading to a drop in strength. Figure 7 depicts the load–displacement curve of typical laser heating power density, from which a similar conclusion can be reached. When the laser heating power density is low (37.1 W/cm^2^), the maximum displacement of the material under tensile load increases compared with that under normal temperature, and the maximum load is also increased; when the laser heating power density increases continuously to 140.6 W/cm^2^, the maximum displacement of the specimen decreases continuously, and the load of the specimen decreases; when the temperature increases further to about 1550 ℃, the load of the specimen increases. Furthermore, the maximum displacement rose substantially. The results indicate that the modulus of the material varies little at high temperatures and that the load-bearing variation is primarily attributable to the decrease in the material's ultimate deformation at high temperatures.

The high-temperature mechanical properties of the material were measured by double-side heating with a flat-top laser beam, which is particularly suitable for testing mechanical properties in an aerobic environment above 600 °C. When the temperature is low, it is preferable to use other heating sources. This approach is particularly suited for aerobic environments because no specific equipment or water-cooling system is required. In the experiment, the maximum temperature rise rate of the laser reaches 260 ℃/s, and all the testing time is controlled within 35 s. If the laser output power density is increased further, the rate of temperature rise will increase. This approach has numerous advantages over the standard high-temperature testing method, including great efficiency and a simple structure. It is expected to be widely used for testing the mechanical properties of ultra-high-temperature materials at high temperatures.

**Figure 8:**
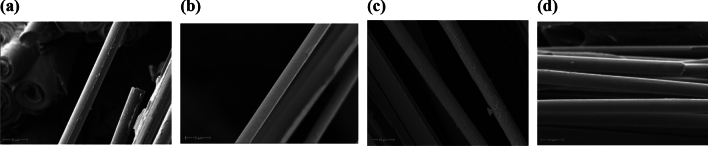
SEM image of the fracture surface of the specimen material after the experiment. (**a**) 37.1 W/cm^2^ (895℃), (**b**) 70.3 W/cm^2^ (1054 ℃), (**c**) 140.6 W/cm^2^ (1470 ℃), (**d**) 281.2 W/cm^2^ (1724 ℃).

After stretching, carbon fibres at the cross-section of specimens heated to different temperatures with different laser power densities were observed using scanning electron microscopy (SEM). Figure 8 shows typical results of carbon fibre damage observed by SEM after heating with four different laser power densities, with the highest temperature reached by laser heating shown in parentheses. The SEM was used to observe the degradation of carbon fibres that primarily support loads on a 10-micron scale. The results indicate that the laser power density is directly proportional to the temperature at which the specimen undergoes fracture. However, the overall structure of the carbon fibre is intact, and there is no significant difference in the surface morphology between the 281.2 W/cm^2^ laser-heated carbon fibre filament and the 37.1 W/cm^2^ heated result. When the laser heating temperature hits 1724 °C, there is still no visible damage on the carbon fibre filament's surface. It implies that there are no evident indicators of carbon fibre ablation and oxidation under laser-induced high temperature and rapid temperature rise. Using laser heating to achieve quick mechanical performance testing of materials at high temperatures eliminates the issue of excessive oxidation at aerobic high temperatures.

## Conclusion

In this paper, the high-temperature tensile strength of C/SiC material at high temperatures was tested by using a laser as a heating source, and the tensile strength at high temperatures was obtained under aerobic conditions. At a high rate of temperature rise, the high-temperature mechanical properties of C/SiC materials are comparable to conventional high-temperature mechanical properties. Because the experimental duration under laser heating is brief and the oxidation factors are low, the mechanical properties are comparable to those determined in an anaerobic environment, whereas the experimental results obtained by long-term heating in an oxygen-free environment are obviously different. In the lack of future experimental results for high-temperature mechanical properties, it is possible to substitute the mechanical properties of materials in an anaerobic environment.

Due to the high heating rate of the laser, the testing time of this method is mainly determined by the tensile speed of the testing machine. In this test, the testing machine selected a faster tensile speed, which proves that this method can be applied to short-term high-temperature mechanical property testing environments, which is not achievable by other testing methods. If the loading speed of the testing machine is slower. The specimen will be heated by the laser and remain at a certain temperature for a long time until it fractures and obtains its strength. At this point, the effect of laser heating is the same as that of a traditional high-temperature furnace. Therefore, this testing method can also be used for traditional high-temperature aerobic mechanical property testing.

## Data Availability

The datasets used and analysed during the current study available from the corresponding author on reasonable request.
